# Stopping sperm in their tracks

**DOI:** 10.7554/eLife.55396

**Published:** 2020-02-27

**Authors:** Luke L McGoldrick, Jean-Ju Chung

**Affiliations:** 1 Department of Cellular and Molecular Physiology, Yale School of MedicineNew HavenUnited States; 2 Department of Obstetrics, Gynecology, and Reproductive Sciences, Yale School of MedicineNew HavenUnited States

**Keywords:** sperm, contraception, motility, infertility, acrosome reaction, high-throughput screening, Human

## Abstract

An automated high-throughput platform can screen for molecules that change the motility of sperm cells and their ability to fertilize.

**Related research article** Gruber FS, Johnston ZC, Barratt CLR, Andrews PD. 2020. A phenotypic screening platform utilising human spermatozoa identifies compounds with contraceptive activity. *eLife* **9**:e51739. doi: 10.7554/eLife.51739

From 2010 to 2014, about 44% of pregnancies worldwide were unplanned, and over half of these ended in abortion in both developed and developing countries (Bearak et al., 2018). Moreover, these terminations frequently result in the death of women in some regions of the world (Ganatra et al., 2017; WHO, 2011). These statistics confirm the need to make effective family planning more widely available to those who want to adopt it, as this brings positive outcomes to women, families, countries and the environment (Starbird et al., 2016).

Precursors to the modern condom were used in ancient Egypt, and hormone-based female oral contraceptives have been used since the middle of the twentieth century (Khan et al., 2013; Dhont, 2010). Today, most contraceptives are for use by women. Traditional male options include condoms, which are only partially effective, and vasectomies, which men may be hesitant to get because they cannot always be reversed (Manetti and Honig, 2010; Gava and Meriggiola, 2019). There is therefore a need for new male contraceptives, with sperm being an obvious target.

Sperm cells are equipped with a tail-like structure that allows them to be motile and to travel through the female reproductive tract to reach the egg. There, they undergo the acrosome reaction: this involves the sperm cell releasing digestive enzymes that allow it to come into close contact and fuse with an egg. Successful fertilization requires both motility and a successful acrosome reaction, so targeting and disrupting these processes is a good strategy to develop effective male contraceptives.

Now, in eLife, Franz Gruber, Zoe Johnston, Christopher Barratt and Paul Andrews at the University of Dundee report having developed a new screening platform to evaluate the effects of small molecules on the motility of sperm cells and their ability to go through the acrosome reaction (Gruber et al., 2020). The team used a collection of about 12,000 clinical or preclinical small molecules for which human safety data are already available.

To evaluate the effect these molecules have on sperm motility, a robotics system was used to incubate each small molecule with separate batches of sperm for 10 minutes (Figure 1); the cells were then imaged using a camera with a high frame rate to capture motion. In total, 63 candidates were identified as reducing motility by 15% or more, with 29 being confirmed when retested. Some of the hits were promising, such as Disulfiram and KF-4939. Disulfiram has been used to treat alcohol dependency and is known to dampen sperm motility (Lal et al., 2016). KF-4939 is thought to inhibit platelet aggregation factors, which have already been used in male infertility treatments to improve sperm motility (Roudebush et al., 2004). The experiment also highlighted a large number of small molecules that increase, rather than decrease, sperm motility: these molecules need to be investigated further as they could be used to treat infertility.

**Figure 1.**
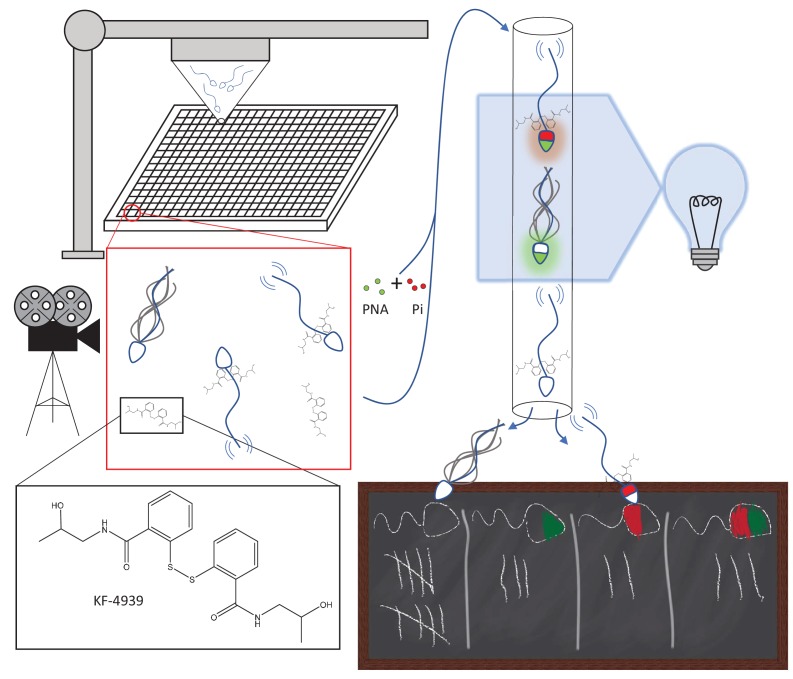
Automated targeting of sperm motility and acrosome reaction with small molecules. Sperm are robotically allocated into wells (~10,000 cells/well) in a 384-well plate (top left); each well contains a different small molecule at a concentration of ~6 μM. The insets show KF-4939, an anti-platelet agent, binding to sperm cells. After incubation with the small molecules, sperm motility is imaged and assessed (middle left). Subsequently, the same sperm are mixed with two tags, peanut agglutinin (PNA; green) and propidium iodide (Pi; red), that emit fluorescence when attached to cells. Sperm were analyzed with a technique called flow cytometry (top right): PNA binding to a sperm cell indicates that the cell has undergone the acrosome reaction, while Pi only binds to dead cells. Most sperm cells do not bind to PNA or Pi, some bind to one but not the other, and some bind to both (bottom right).

To improve the efficiency of the screen, the same cells that had been assessed for motility were also examined for the acrosome reaction. Two fluorescent tags, called peanut agglutinin and propidium iodide, helped to detect sperm cells that had gone through the reaction after being exposed to the small molecules. Peanut agglutinin only binds to the heads of sperm cells that have undergone the acrosome reaction, and propidium iodide exclusively attaches to dead cells. An automated technique called flow cytometry was used to record whether the tags were bound to a given cell, therefore measuring the percentage of live cells that had undergone the acrosome reaction after being exposed to the small molecules. However, all nine hits proved to be false positives as the small molecules themselves were fluorescent, highlighting a need to carry on such screenings while being wary of artefacts.

The high-throughput screening platform developed by Gruber et al. has the potential to make important contributions to the search for contraceptives and male infertility drugs. This work only examined one collection of molecules, and two sperm parameters, motility and acrosome reaction. Going forward, it would be interesting to apply this platform to target other aspects of sperm biology that are critical for fertilization, such as hyperactivated motility (when sperm cells vigorously and asymmetrically wag their tail-like structures to enter the egg), or the binding of sperm cells to specific egg proteins.
